# Butane-1,2,3,4-tetra­carboxylic acid dihydrate

**DOI:** 10.1107/S1600536809009970

**Published:** 2009-03-25

**Authors:** Yu Cheng, Jiang Wu, Hong-Lin Zhu, Jian-li Lin

**Affiliations:** a State Key Laboratory Base of Novel Functional Materials and Preparation Science, Faculty of Materials Science and Chemical Engineering, Institute of Solid Materials Chemistry, Ningbo University, Zhejiang 315211, People’s Republic of China

## Abstract

The asymmetric unit of the title compound, C_8_H_10_O_8_·2H_2_O, contains one half-mol­ecule of butane-1,2,3,4-tetra­carboxylic acid and a water mol­ecule, with the complete tetra-acid generated by crystallographic inversion symmetry. Inter­molecular O—H⋯O hydrogen bonds form an extensive three-dimensional network, which consolidates the crystal packing.

## Related literature

For applications of butane-1,2,3,4-tetra­carboxylic acid in metal -organic coordination polymers, see: Delgado *et al.* (2007 ▶); Liu *et al.* (2008 ▶). For related crystal structures, see: McKee *et al.* (2007 ▶); Najafpour *et al.* (2008 ▶).
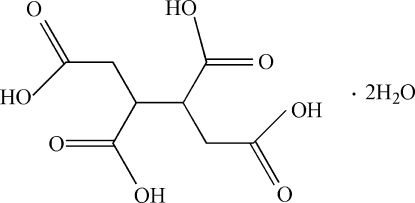


## Experimental

### 

#### Crystal data

C_8_H_10_O_8_·2H_2_O
*M* *_r_* = 270.19
Monoclinic, 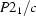

*a* = 7.4668 (15) Å
*b* = 9.3385 (19) Å
*c* = 8.8406 (18) Å
β = 109.60 (3)°
*V* = 580.7 (2) Å^3^
*Z* = 2
Mo *K*α radiation
μ = 0.15 mm^−1^
*T* = 293 K
0.55 × 0.46 × 0.26 mm

#### Data collection

Rigaku R-AXIS RAPID diffractometer
Absorption correction: multi-scan (*ABSCOR*; Higashi, 1995 ▶) *T*_min_ = 0.921, *T*_max_ = 0.965
5478 measured reflections
1327 independent reflections
960 reflections with *I* > 2σ(*I*)
*R*_int_ = 0.027

#### Refinement

*R*[*F* ^2^ > 2σ(*F* ^2^)] = 0.039
*wR*(*F* ^2^) = 0.116
*S* = 1.17
1327 reflections
82 parameters
H-atom parameters constrained
Δρ_max_ = 0.26 e Å^−3^
Δρ_min_ = −0.23 e Å^−3^

### 

Data collection: *RAPID-AUTO* (Rigaku, 1998 ▶); cell refinement: *RAPID-AUTO*; data reduction: *CrystalStructure* (Rigaku/MSC, 2004 ▶); program(s) used to solve structure: *SHELXS97* (Sheldrick, 2008 ▶); program(s) used to refine structure: *SHELXL97* (Sheldrick, 2008 ▶); molecular graphics: *SHELXTL* (Sheldrick, 2008 ▶); software used to prepare material for publication: *SHELXL97*.

## Supplementary Material

Crystal structure: contains datablocks global, I. DOI: 10.1107/S1600536809009970/cv2528sup1.cif

Structure factors: contains datablocks I. DOI: 10.1107/S1600536809009970/cv2528Isup2.hkl

Additional supplementary materials:  crystallographic information; 3D view; checkCIF report

## Figures and Tables

**Table 1:** Hydrogen-bond geometry (Å, °)

*D*—H⋯*A*	*D*—H	H⋯*A*	*D*⋯*A*	*D*—H⋯*A*
O2—H2*C*⋯O5^i^	0.85	1.87	2.707 (2)	167
O4—H4*A*⋯O5^ii^	0.86	1.83	2.689 (2)	178
O5—H5*A*⋯O3	0.83	1.93	2.754 (2)	172
O5—H5*B*⋯O1^iii^	0.81	2.01	2.814 (2)	170

Symmetry codes: (i) 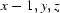; (ii) 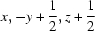; (iii) 
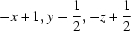
.

**Fig. 1.**
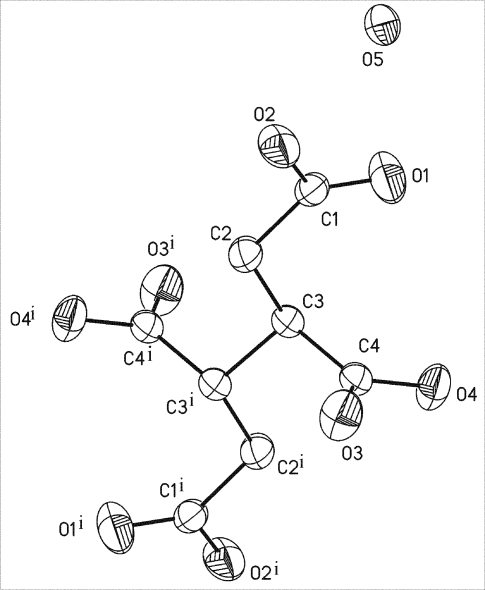
View of the title compound showing the atomic numbering and 45% probability dispalcement ellipsoids [symmetry code: (i) -x + 1, -y + 1, -z]. H atoms omitted for clarity.

## supplementary crystallographic information

### Comment

A search of the Cambridge Structural Database (Version 5.30, February 2009) showed that most of literature dealing with butane-1,2,3,4-tetracarboxylic acid mainly concentrated in the metal organic coordination polymers (Delgado *et al.*, 2007; Liu *et al.*, 2008). In this paper, we report the crystal structure of butane–1,2,3,4–tetracarboxylic acid dihydrate (Fig. 1).

The asymmetric unit of the title compound contains a half of the butane-1,2,3,4-tetracarboxylic acid molecule and one water molecule. The carboxylic acid group with C1 and C4 atoms are *gauche* with the C1—C2—C3—C4 torsion angle being 62.13 (1)°, which match well with that in the reported structures (McKee *et al.*, 2007; Najafpour *et al.*, 2008). Intermolecular O—H···O hydrogen bonds (Table 1) form an extensive three-dimensional hydrogen-bonding network, which consolidate the crystal packing.

### Experimental

Zn(NO_3_)_2_.6H_2_O (0.1461 g, 1.0 mmol) was added to a stirred aqueous solution of butane-1,2,3,4-tetracarboxylic acid (0.1176 g, 0.50 mmol) in 15 ml H_2_O, the resulting mixture was stirred for 20 min and then was filtered out. Colorless crystals were obtained from the filtrate (pH=2.80) after standing at room temperature for three months.

### Refinement

H atoms bonded to C atoms were palced in geometrically calculated position and were refined using a riding model, with *U*_iso_(H) = 1.2 *U*_eq_(C). H atoms attached to O atoms were found in a difference Fourier synthesis and were refined using a riding model, with the O—H distances fixed as initially found and with *U*_iso_(H) values set at 1.5 *U*eq(O).

### Crystal data

**Table:**

C_8_H_10_O_8_·2H_2_O	*F*(000) = 284
*M**_r_* = 270.19	*D*_x_ = 1.545 Mg m^−^^3^
Monoclinic, *P*2_1_/*c*	Mo *K*α radiation, λ = 0.71073 Å
Hall symbol: -P 2ybc	Cell parameters from 5478 reflections
*a* = 7.4668 (15) Å	θ = 3.3–27.4°
*b* = 9.3385 (19) Å	µ = 0.15 mm^−^^1^
*c* = 8.8406 (18) Å	*T* = 293 K
β = 109.60 (3)°	Platelet, colorless
*V* = 580.7 (2) Å^3^	0.55 × 0.46 × 0.26 mm
*Z* = 2	

### Data collection

**Table:**

Rigaku R-AXIS RAPID diffractometer	1327 independent reflections
Radiation source: fine-focus sealed tube	960 reflections with *I* > 2σ(*I*)
graphite	*R*_int_ = 0.027
Detector resolution: 0 pixels mm^-1^	θ_max_ = 27.4°, θ_min_ = 3.3°
ω scans	*h* = −9→9
Absorption correction: multi-scan (*ABSCOR*; Higashi, 1995)	*k* = −12→12
*T*_min_ = 0.921, *T*_max_ = 0.965	*l* = −11→11
5478 measured reflections	

### Refinement

**Table:**

Refinement on *F*^2^	Primary atom site location: structure-invariant direct methods
Least-squares matrix: full	Secondary atom site location: difference Fourier map
*R*[*F*^2^ > 2σ(*F*^2^)] = 0.039	Hydrogen site location: inferred from neighbouring sites
*wR*(*F*^2^) = 0.116	H-atom parameters constrained
*S* = 1.17	*w* = 1/[σ^2^(*F*_o_^2^) + (0.0345*P*)^2^ + 0.3695*P*] where *P* = (*F*_o_^2^ + 2*F*_c_^2^)/3
1327 reflections	(Δ/σ)_max_ < 0.001
82 parameters	Δρ_max_ = 0.26 e Å^−^^3^
0 restraints	Δρ_min_ = −0.23 e Å^−^^3^

### Special details

**Table:**

Geometry. All e.s.d.'s (except the e.s.d. in the dihedral angle between two l.s. planes) are estimated using the full covariance matrix. The cell e.s.d.'s are taken into account individually in the estimation of e.s.d.'s in distances, angles and torsion angles; correlations between e.s.d.'s in cell parameters are only used when they are defined by crystal symmetry. An approximate (isotropic) treatment of cell e.s.d.'s is used for estimating e.s.d.'s involving l.s. planes.
Refinement. Refinement of *F*^2^ against ALL reflections. The weighted *R*-factor *wR* and goodness of fit *S* are based on *F*^2^, conventional *R*-factors *R* are based on *F*, with *F* set to zero for negative *F*^2^. The threshold expression of *F*^2^ > σ(*F*^2^) is used only for calculating *R*-factors(gt) *etc*. and is not relevant to the choice of reflections for refinement. *R*-factors based on *F*^2^ are statistically about twice as large as those based on *F*, and *R*- factors based on ALL data will be even larger.

### Fractional atomic coordinates and isotropic or equivalent isotropic displacement parameters (Å^2^)

**Table:**

	*x*	*y*	*z*	*U*_iso_*/*U*_eq_	
O1	0.1296 (3)	0.38628 (19)	0.1509 (2)	0.0561 (5)	
O2	0.0636 (2)	0.20225 (18)	−0.0167 (2)	0.0494 (5)	
H2C	−0.0132	0.1808	0.0327	0.074*	
C1	0.1546 (3)	0.3207 (2)	0.0422 (2)	0.0319 (5)	
C2	0.2916 (3)	0.3636 (2)	−0.0416 (2)	0.0371 (5)	
H2A	0.2201	0.3991	−0.1479	0.044*	
H2B	0.3613	0.2795	−0.0547	0.044*	
C3	0.4336 (3)	0.4784 (2)	0.0479 (2)	0.0294 (4)	
H3A	0.3640	0.5630	0.0633	0.035*	
C4	0.5554 (3)	0.4210 (2)	0.2107 (2)	0.0299 (4)	
O3	0.6322 (3)	0.30568 (17)	0.23027 (19)	0.0510 (5)	
O4	0.5724 (2)	0.50976 (17)	0.33017 (16)	0.0454 (4)	
H4A	0.6470	0.4737	0.4186	0.068*	
O5	0.8064 (2)	0.09678 (15)	0.10900 (16)	0.0369 (4)	
H5A	0.7639	0.1616	0.1518	0.055*	
H5B	0.8321	0.0309	0.1725	0.055*	

### Atomic displacement parameters (Å^2^)

**Table:**

	*U*^11^	*U*^22^	*U*^33^	*U*^12^	*U*^13^	*U*^23^
O1	0.0693 (12)	0.0600 (11)	0.0536 (10)	−0.0267 (9)	0.0398 (9)	−0.0219 (8)
O2	0.0483 (9)	0.0517 (10)	0.0578 (10)	−0.0243 (8)	0.0303 (8)	−0.0182 (8)
C1	0.0289 (10)	0.0377 (11)	0.0272 (9)	−0.0048 (8)	0.0069 (8)	0.0003 (8)
C2	0.0337 (10)	0.0477 (13)	0.0309 (10)	−0.0117 (9)	0.0124 (8)	−0.0073 (9)
C3	0.0267 (9)	0.0347 (11)	0.0286 (9)	−0.0025 (8)	0.0116 (8)	−0.0012 (8)
C4	0.0275 (9)	0.0348 (11)	0.0294 (9)	−0.0040 (8)	0.0122 (8)	−0.0023 (8)
O3	0.0669 (11)	0.0366 (9)	0.0451 (9)	0.0151 (8)	0.0126 (8)	−0.0019 (7)
O4	0.0549 (10)	0.0470 (9)	0.0278 (7)	0.0170 (7)	0.0054 (7)	−0.0057 (6)
O5	0.0434 (8)	0.0352 (8)	0.0358 (7)	−0.0005 (6)	0.0182 (6)	0.0018 (6)

### Geometric parameters (Å, °)

**Table:**

O1—C1	1.206 (2)	C3—C3^i^	1.559 (3)
O2—C1	1.311 (2)	C3—H3A	0.9800
O2—H2C	0.8523	C4—O3	1.205 (2)
C1—C2	1.505 (3)	C4—O4	1.315 (2)
C2—C3	1.528 (3)	O4—H4A	0.8618
C2—H2A	0.9700	O5—H5A	0.8314
C2—H2B	0.9700	O5—H5B	0.8111
C3—C4	1.518 (3)		
			
C1—O2—H2C	110.1	C4—C3—C3^i^	108.55 (18)
O1—C1—O2	123.13 (18)	C2—C3—C3^i^	110.94 (19)
O1—C1—C2	124.71 (18)	C4—C3—H3A	109.3
O2—C1—C2	112.15 (17)	C2—C3—H3A	109.3
C1—C2—C3	113.57 (16)	C3^i^—C3—H3A	109.3
C1—C2—H2A	108.9	O3—C4—O4	122.30 (18)
C3—C2—H2A	108.9	O3—C4—C3	123.80 (18)
C1—C2—H2B	108.9	O4—C4—C3	113.89 (17)
C3—C2—H2B	108.9	C4—O4—H4A	110.0
H2A—C2—H2B	107.7	H5A—O5—H5B	105.9
C4—C3—C2	109.54 (16)		

Symmetry codes: (i) −*x*+1, −*y*+1, −*z*.

### Hydrogen-bond geometry (Å, °)

**Table:**

*D*—H···*A*	*D*—H	H···*A*	*D*···*A*	*D*—H···*A*
O2—H2C···O5^ii^	0.85	1.87	2.707 (2)	167
O4—H4A···O5^iii^	0.86	1.83	2.689 (2)	178
O5—H5A···O3	0.83	1.93	2.754 (2)	172
O5—H5B···O1^iv^	0.81	2.01	2.814 (2)	170

Symmetry codes: (ii) *x*−1, *y*, *z*; (iii) *x*, −*y*+1/2, *z*+1/2;  (iv) −*x*+1, *y*−1/2, −*z*+1/2.

## References

[bb1] Delgado, L. C., Fabelo, O., Pasàn, J., Delgado, F. S., Lloret, F., Julve, M. & Ruiz-Pérez, C. (2007). *Inorg. Chem.* **46**, 7458–7465. 10.1021/ic7006765 17691768

[bb2] Higashi, T. (1995). *ABSCOR* Rigaku Corporation, Tokyo, Japan.

[bb3] Liu, Y. Y., Ma, J. F., Yang, J., Ma, J. C. & Su, Z. M. (2008). *CrystEngComm*, **10**, 894–904.

[bb4] McKee, V. & Najafpour, M. M. (2007). *Acta Cryst.* E**63**, o741–o743.

[bb5] Najafpour, M. M., Hołyńska, M. & Lis, T. (2008). *Acta Cryst.* E**64**, o985. 10.1107/S1600536808011732 PMC2961488 21202711

[bb6] Rigaku (1998). *RAPID-AUTO* Rigaku Corporation, Tokyo, Japan.

[bb7] Rigaku/MSC (2004). *CrystalStructure* Rigaku/MSC Inc., The Woodlands, Texas, USA.

[bb8] Sheldrick, G. M. (2008). *Acta Cryst.* A**64**, 112–122. 10.1107/S0108767307043930 18156677

